# Exploratory serum fatty acid patterns associated with blood pressure in community-dwelling middle-aged and elderly Chinese

**DOI:** 10.1186/s12944-016-0226-3

**Published:** 2016-03-18

**Authors:** Bo Yang, Fang Ding, Jing Yan, Xiong-Wei Ye, Xiao-Lin Xu, Feng-Lei Wang, Duo Li, Wei Yu

**Affiliations:** Department of Food Science and Nutrition, Zhejiang University, 866 Yuhangtang Road, Hangzhou, 310058 China; The Province Center for Cardio-Cerebral-Vascular Disease, Zhejiang Hospital, 12 Linyin Road, Hangzhou, 310013 China

**Keywords:** Blood pressure, Fatty acid, Factor analysis, Hypertension

## Abstract

**Background:**

Epidemiological studies have assessed relationships between circulating levels of fatty acid (FA) and blood pressure (BP), and their results remain controversial. Nevertheless, data are sparse on serum FA as biomarker and BP in China. The aim of the study was to investigate the association between serum FA and BP in Chinese populations.

**Methods:**

We conducted a cross-sectional study nested within a community-based cohort of 2447 Chinese participants aged 35–79 years who completed a baseline assessment between October 2012 and April 2013. Baseline assessment included the collection of fasting blood samples, anthropometric measurements and a personal interview using a validated questionnaire. Serum FA was determined by gas-liquid chromatography. Exploratory factor analyses were employed to identify FA-factor as a reflection of serum FA pattern. A multiple regression model was conducted to estimate adjusted mean of BP with 95 % confidence interval (CI) by tertile groups of the generated FA-factor scores.

**Results:**

Hypertensive patients have significantly higher serum 14:0, 16:0, 16:1n-7, 18:3n-6, 20:3n-6 and Δ^6^-desaturase index (18:3n-6/18:2n-6) as well as lower 18:2n-6, 22:6n-3 and Δ^5^-desaturase index (20:4n-6/20:3n-6) compared with normotensive participants. Factor 1 (low linoleic acid/high saturated FA pattern: 14:0, 16:0, 16:1n-7, 18:2n-6, 18:3n-6, 20:3n-6) and Factor 2 (n-3 PUFA pattern: 20:5n-3, 22:5n-3, 22:6n-3, 18:1n-9) were identified as indicators of the serum FA pattern. After adjustment for age, gender, body mass index, hypertension treatment, smoking, alcohol intake, education, profession, exercise habit, salt intake, family history of hypertension, heart rate, blood lipids and fasting blood-glucose levels, per a standard deviation (SD) increment of Factor 1 scores was associated with an increment of 2.44 (95 % CI: 1.73, 3.15) mm Hg for systolic BP, whereas per a SD increment of Factor 2 scores was associated with a reduction of 1.40 (95 % CI: 0.80, 2.04) mm Hg for diastolic BP.

**Conclusions:**

The serum FA pattern characterized by low proportions of 14:0, 16:0, 16:1n-7 and 18:3n-6 as well as high 18:2n-6 and 22:6n-3 was beneficially associated with BP levels in this Chinese population. This evidence well supports the current dietary recommendations in the communities to replace saturated fat with polyunsaturated fat.

**Electronic supplementary material:**

The online version of this article (doi:10.1186/s12944-016-0226-3) contains supplementary material, which is available to authorized users.

## Background

Adverse blood pressure (BP) levels remain a major public-health problem worldwide, and almost 2.11 million cardiovascular deaths are attributable to greater BP in China annually [1]. Data from meta-analyses [2, 3] and clinical trials [4, 5] have suggested that n-3 polyunsaturated fatty acid (PUFA) supplement can dose-dependently lower BP in hypertensive patients but not in normotensive individuals. A large-scale, population-based International Study of Macro/Micronutrients and Blood Pressure (INTERMAP) suggested that dietary intake of n-3 PUFA was inversely associated with BP levels in middle-aged normotensive persons [6]. However, its potential limitations are likely to be dietary measurement errors or bias with consequent limited ability to classify dietary intake of individuals accurately and underestimation of effect size attributable to limited reliability in dietary estimations.

In contrast to dietary questionnaires, FA levels in circulating blood can provide objective measures that reflect both dietary consumption and relevant biological processes. Additionally, FA biomarker can also permit direct evaluation of individual FA that may have different effects on certain biological pathways or clinical end points. Examples of this have been reported that circulating levels of PUFA were significantly correlated with intake of polyunsaturated fat [7], while circulating levels of 14:0, 16:0, 18:0 and monounsaturated FA (MUFA) may reflect a diet rich in saturated fat, because of their high correlations with saturated fat [8]. Prior observational studies examined associations between serum/plasma FA as biomarker and BP, and their finding were controversial [9–11]. Possible reasons for the inconsistencies across studies may be BP measurements insufficiently standardized and small sample sizes with a low statistical power. Recently, a large-scale longitudinal study of erythrocyte FA in relation to BP change in 1834 middle-aged and elderly Chinese individuals indicated that erythrocyte long-chain (LC) n-3 PUFA was associated reduced BP levels of individuals [12]. However, the small changes in BP and relatively great errors in BP measurements during follow-up would substantially attenuate the FA-BP associations. Additionally, the amount of each FA is often expressed as a proportion and is a relative measure, which means that many of individual FA are inter-related. A change in the proportion of any specific FA will influence the proportions of several others in blood, which was likely to bias the true association between individual FA and BP. Thus, the association between circulating levels of FA as biomarker and BP of individuals merit further study.

We therefore attempted to employ a method of exploratory factor analyses to identify the specific FA-factors as an indicator of FA patterns [13]. Totally, 2447 community-dwellers (1153 males and 1294 females) aged 35–79 years living in Zhejiang Province, southeast China were recruited through a cross-sectional study nested with in a community-based cohort. The aim of the study was to investigate the association between serum FA patterns and BP levels in this middle-aged and elderly Chinese population.

## Methods

### Study design

The cross-sectional study with baseline data was conduced within a community-based cohort (Zhejiang Prospective Investigation into Hypertension and Lifestyle, ZPIHL). The study protocol was approved by the Ethics Committee, Zhejiang Hospital, China. All subjects provided their written consent prior to participation in the study. The detailed methods for participant selection are presented elsewhere [14]. Briefly, study participants were recruited from 6 community health centers across 12 counties located in Zhejiang Province, southeast China. Zhejiang province is a well development region with a good medicare system even in remote areas. Totally, 3,500 individuals aged 35–79 years were invited between April and October 2012. Participants are all local Chinese residents, and their complete healthy/medical records were documented in the local community hospitals. To define study participants, we excluded anyone who has no complete medical record, and meet any of the following items: myocardial infarction, coronary heart disease, peripheral vascular disease, angina, cancer, dementia, schizophrenia, deaf or dumb, < 35 years, less than 6 months of living in the local communities. Finally, 3,017 participants with complete health records agreed to participate in the study. Baseline assessment included the collection of fasting blood samples, anthropometric measurements and a personal interview using a validated questionnaire about medical history, socio-demographic and lifestyle characteristics between October 2012 and April 2013. Of those, 2,535 subjects provided fasting blood samples and completed a laboratory assessment, with a participation rate of 84.02 % (2535/3017). Subjects with missing values (*n* = 56) or implausible data (*n* = 19) on lifestyle factors from the structured questionnaire and with unreliable data on serum FA (*n* = 13) were excluded. Afterwards, 2,447 participants (1,153 males and 1,294 females) were finally remained for baseline analyses.

### Questionnaire interview

A face-to-face interview was conducted by trained interviewers using a validated questionnaire. We collected data on socio-demographic, medical history, and lifestyle factors. Education levels were categorized as primary (duration of education < 9 years), secondary (9–12 years) and high (>12 years). High-salt intake was defined as salt consumption with > 6 g per day [15]. Regular exercisers were those who sported ≥ 3 times per week for over 6 months. Current smokers were those who smoked ≥18 packs per year, while current drinkers were those who consumed alcoholic beverage ≥ 25 g per day.

### Measurements of anthropometric parameters and blood pressure

Anthropometrical measurements were performed by trained nurses using standard protocols. Body weight (kg) and height (m) were measured with participants wearing no shoes and light clothing. Body mass index (BMI) was calculated as the participant’s weight (kg) divided by the square of standing height (m). Before heart rate (HR, beat/min) and blood pressure (BP, mm Hg) measurements**,** participants were advised to avoid consuming alcohol or tobacco, ingesting tea or coffee, and engaging in exercise for at least 30 min. A standardized mercury sphygmomanometer was used to measure BP by trained physicians, and one of three cuff sizes (regular adult, large or thigh) was chosen based on the circumference of the participant’s arm. BP and HR for each subject were defined as the average of three measurements performed in the subject in sitting position with 2-min intervals at that visit. Prevalent hypertension was ascertained by meeting 1 of 2 criteria: (a) a new physician diagnosis of hypertension (an average systolic BP (SBP) ≥ 140 mm Hg and/or an average diastolic BP (DBP) ≥ 90 mmHg); or (b) self-reported treatment of hypertension with anti-hypertensive drugs.

### Laboratory assessment

Fasting blood samples were collected in tubes with serum separator gel. They were left at room temperature for 30 min and then centrifuged at 2500 RCF (g) for 15 mins to isolate serum. Measurement of fasting serum triglycerides (TG), total cholesterol (TC) and glucose (Fbg) levels were determined by standard procedures at the biochemistry laboratory of Zhejiang Hospital. The remaining serum samples were aliquoted into separate tubes (1 mL) and stored at -80 ^0^C until further FA analyses.

Frozen samples were thawed on ice for 40 mins. The total lipid content of the serum was extracted with solvents. The methyl esters of FA in serum were prepared by saponification, and serum compositions of FA were determined by gas-liquid chromatography (GLC) as described previously [16]. The main sources of serum FA are derived from cholesterol esters, phospholipids, and triglycerides. The amount of individual FA was expressed as a percentage of total FA in serum.

### Statistical analysis

Statistical analyses of all the data were performed by STATA version 11.0 (Stata CORP, College Station, TX). Descriptive statistics was initially conducted. The distribution of continuous variables was examined for normality by Shapiro-Wilk’s test. Continuous data with the normal distribution were expressed as the mean (standard deviation), while categorical data were expressed as a proportion. The skewed data were expressed as the median (quartile range), and were log-transformed before statistic analyses. Difference in the continuous and categorical variables between two groups was examined by Student’s *t*-test and chi-square test, respectively. Factor analysis has been a multivariate statistical tool of dimension-reduction to study FA patterns [13], which can be performed to address inter-relations between individual FA content. Individual FA was entered in a factor analysis model followed by variance rotation to identify the specific FA-factors as a measure of FA pattern. The resulting FA-factor pattern was determined by a factor loading, the eigenvalue and the explained proportion of total variance. Factor loading of each individual FA equals the Pearson correlation coefficient between that FA and the generated FA-factor. Factor loading with ≥ |0.40| were considered meaningful for the interpretation of FA-factor [17]. Eigenvalue described the amount of variance attributable to the FA-factors. The powerful FA-factors with eigenvalue > 1 were extracted for further analyses, which explained more than 70 % of the total variance. Factor scores for each FA pattern can be calculated as a sum of the products of factor loading coefficients and the standardized each FA correlated with that FA pattern. To determine if serum FA patterns were associated with BP levels, a multiple regression model was conducted to estimate adjusted mean of BP with 95 % confidence interval (CI) by tertile groups of the generated FA-factor scores. The ordinal numbers 0–2 was assigned to the tertile (T) categories of FA-factor scores, and then a linear trend test was performed by treating the ordinal score variable as a continuous variable in the models. The dependent variables (SBP and DBP), biochemical parameters (TC, TG, and Fbg levels) and anthropometrical data (HR and BMI) were continuous, while socio-demographic (age (≤55, >55), gender, profession (manual, non-manual), and education levels), lifestyle data (smoking habit (current, former/never), alcohol consumption (current, former/never), high-salt diet (yes, no)) and family history of hypertension (yes, no) as the independent variables were categorical. Initial model was adjusted for age, gender, BMI and hypertension treatment; model 1 was adjusted for the covariates in model 1 plus lifestyle factors; model 2 as a full model was adjusted for the covariates in model 2 plus clinical parameters (family history of hypertension, TC, TG, HR and Fbg levels). In addition, to test the robustness of the findings from factor analyses, a multiple regression analysis was repeated to assess associations of individual FA contributing most to FA-factors with BP levels. All statistic tests were two-sided, and *P* < 0.05 was considered statistically significant.

## Results

### Baseline characteristics

Totally, 748 hypertensive patients were identified from 2447 community-dwellers aged 35–79 years who completed baseline measurements. Characteristics of study participants according to BP status are presented in Table 1. All participants are comprised of 1153 males (47.12 %) and 1294 females (52.88 %). The proportion of prevalent hypertension was significantly higher in 1,201 middle-aged than in 1,246 elderly participants. Hypertensive patients likely tended to have primary education levels, non-manual labor, high-salt diet, irregular/never sporters, family history of hypertension, higher BMI, BP, HR, TG, TC and Fbg levels compared with normotensive participants (all *P* < 0.05).

**Table 1:** Characteristics of participants by BP status in Zhejiang Province, China

Variables	Total subjects (*n* = 2447)	Hypertensive (*n* = 748)	Normotensive (*n* = 1699)	*P* ^a^
Demographics, %^b^				
Age,				< 0.01
Middle-age (≤ 55 y)	49.11	53.59	47.13	
Elder (> 55 y)	50.89	46.41	52.87	
Females	52.88	51.87	53.33	0.48
Education levels				< 0.01
Primary (< 9 years)	66.89	75.61	62.31	
Secondary (9–12 years)	27.86	21.41	31.25	
High (>12 years)	5.25	2.98	6.44	
Profession				0.01
Manual labor	38.66	34.24	40.64	
non-manual labor	61.34	65.76	59.36	
Lifestyle factors, %^b^				
Smoking habit				0.01
Current smokers (≥ 18 pack/year)	24.28	20.94	25.75	
Former/never	75.72	79.06	74.25	
Drinking habit				0.04
Current (≥ 25 g/day)	26.86	25.13	28.62	
Former/never	73.14	74.87	71.38	
Salt consumption				0.01
High-salt (> 6 g/day)	25.72	29.21	24.18	
Moderate/low-salt	74.28	70.79	76.82	
Exercise habit				< 0.01
Regular (≥ 3 times/week)	23.85	20.76	28.95	
Irregular/never	76.15	79.24	71.05	
Family history of hypertension, %^b^				
Yes	24.43	34.46	20.99	< 0.01
No	75.57	65.54	79.01	
Anthropometry indicators, mean (SD)^c^				
BMI (kg/m^2^)	23.80 (3.23)	24.93 (3.43)	23.44 (3.18)	< 0.01
SBP (mm Hg)	126.42 (17.95)	137.10 (12.76)	119.44 (14.78)	< 0.01
DBP (mm Hg)	81.22 (7.53)	86.28 (8.66)	79.70 (6.10)	< 0.01
HR (beat/min)	71.00 (10.19)	72.88 (11.07)	70.53 (9.75)	< 0.01
Biochemical parameters, median (QR)^d^				
TG(mmol/L)	1.26 (0.88–1.86)	1.47 (1.01–2.08)	1.19 (0.83–1.73)	< 0.01
TC (mmol/L)	4.59 (4.07–5.24)	4.73 (4.19–5.35)	4.54 (4.02–5.85)	< 0.01
Fbg (mmol/L)	4.64 (4.29–5.08)	4.73 (4.36–5.22)	4.60 (4.24–5.00)	< 0.01

*SD* standard deviation, *BMI* body mass index, *SBP* systolic blood pressure, *DBP* diastolic blood pressure, *HR* heart rate, *QR* quartile range, *TG* triglyceride, *TC* total cholesterol, *Fbg* fasting blood-glucose

^a^ *P* value for significance between hypertensive and normotensive group with Student’s unpaired ttest and chi-square test, respectively

^b^Categorical data are presented as a percentage (%)

^c^Continuous data with a normal distribution are presented as mean (standard deviation)

^d^The skewed data were expressed as the median (quartile range), and were log-transformed before statistic analyses

### Serum fatty acid profile between hypertensive and normotensive participants

Difference in individual FA between hypertension patients and normotensive persons was presented in Table 2. Compared with normotensive participants, hypertension patients had significantly higher proportions of serum 14:0, 16:0, 18:0, 22:0,16:1n-7, 18:1n-9, 18:3n-6, 20:3n-6 and Δ^6^-desaturase index (18:3n-6/18:2n-6) as well as lower 18:2n-6, 22:6n-3 and Δ^5^-desaturase index (20:4n-6/20:3n-6) (*P* < 0.05, respectively). In addition, lower proportions of total n-3 PUFA, total PUFA and n-3/n-6 ratio as well as higher total SFA and SFA/PUFA ratio in serum were observed in the hypertension patients than in the normotensive participants (*P* < 0.05, respectively).

**Table 2:** Serum fatty acid profile between hypertensive and normotensive participants

FA (%)	Hypertensive (*n* = 748)		Normotensive (*n* = 1699)		*P* value^a^
	Mean/Median	SD/QR	Mean/Median	SD/QR	
14:0	1.61	1.40–1.80	1.56	1.29–1.74	0.033
16:0	20.62	2.60	21.05	2.57	<0.001
18:0	6.40	0.85	6.34	0.78	0.036
20:0	0.48	0.30–0.73	0.51	0.20–0.85	0.148
22:0	0.55	0.33–1.43	0.44	0.27–1.27	0.012
24:0	0.39	0.31–0.42	0.40	0.27–0.45	0.127
16:1n-7	1.69	0.82	1.52	0.78	<0.001
18:1n-9	19.98	3.75	18.95	3.75	<0.001
24:1n-9	1.20	0.92–1.34	1.18	0.82–1.22	0.121
18:3n-3	0.94	0.37	0.93	0.41	0.058
20:5n-3	3.31	1.55	3.35	1.60	0.436
22:5n-3	0.47	0.38–0.58	0.46	0.38–0.56	0.528
22:6n-3	1.65	0.51	1.74	0.69	0.005
18:2n-6	27.40	5.21	28.52	5.28	<0.001
18:3n-6	0.36	0.25–0.51	0.32	0.20–0.46	<0.001
20:3n-6	1.16	0.32	1.13	0.31	0.018
20:4n-6	6.04	1.56	6.01	1.62	0.120
20:4n-6/20:3n-6	5.20	0.87	5.66	0.96	<0.001
18:2n-6/18:3n-6 (×10^−2^)	1.34	0.84–2.05	1.13	0.73–1.77	<0.001
SFA	30.69	3.25	28.87	2.62	<0.001
MUFA	21.38	4.26	21.25	4.44	0.504
PUFA	42.25	4.43	43.66	5.41	<0.001
n-3 PUFA	6.17	5.60–7.04	6.28	5.48–7.30	0.024
n-6 PUFA	36.04	5.00	37.37	4.60	0.010
n-3/n-6	0.15	0.13–0.19	0.16	0.13–0.23	0.031
SFA/PUFA	0.81	0.63–0.92	0.68	0.60–0.82	<0.001

*QR* quartile range, *SFA* saturated fatty acid, *MUFA* monounsaturated fatty acid, *PUFA* polyunsaturated fatty acid

^a^ *P* value for difference between groups was tested by an unpaired *t*-test

### Factor analysis of individual FA

Serum compositions of individual FA and rotated factor loadings of the extracted FA-factors were presented in Table 3. The two major FA-factors can be interpreted as a reflection of serum FA patterns, which explained about 68.73 % of individual FA-related variance. Factor 1 as the most powerful factor (eigenvalue = 3.20), termed “low linoleic acid (LA)/high SFA pattern”, comprised a strongly negative loading from 18:2n-6 (linoleic acid) and higher positive loadings from14:0, 16:0, 16:1n-7, 18:1n-9 and 18:3n-6. These non-essential FA can be synthesized endogenously in response to a high intakes of saturated fat. [8] Factor 2 was defined as “n-3 PUFA pattern” (eigenvalue = 1.95), which mainly comprised higher positive loadings from 20:5n-3, 22:5n-3 and 22:6n-3 as well as a strongly negative loading from 18:1n-9.

**Table 3:** Serum proportions of individual fatty acid and varimax rotation loadings of the major FA-factors

Fatty acid (FA)	Proportions^a^	Factor 1	Factor 2
14:0	1.69 (1.43–1.95)	**0.39** ^b^	−0.06
16:0	20.76 (2.60)	**0.64** ^b^	−0.04
18:0	6.42 (0.89)	0.10	0.13
20:0	0.45 (0.29–0.73)	−0.04	0.29
22:0	0.41 (0.25)	−0.28	0.01
24:0	0.36 (0.11)	−0.26	0.13
16:1n-7	1.59 (0.84)	**0.79** ^b^	0.16
18:1n-9	19.30 (3.83)	0.35	−**0.46** ^c^
24:1n-9	1.04 (0.76–1.38)	−0.02	−0.02
18:2n-6	28.26 (5.31)	−**0.86** ^b^	−0.19
18:3n-6	0.34 (0.22–0.48)	**0.46** ^b^	0.31
20:3n-6	1.14 (0.31)	**0.39** ^b^	0.29
20:4n-6	6.02 (1.61)	−0.25	0.37
18:3n-3	0.93 (0.37)	0.17	−0.23
20:5n-3	3.25 (1.23)	0.03	**0.55** ^c^
22:5n-3	0.51 (0.24)	0.31	**0.44** ^c^
22:6n-3	1.73 (0.62)	−0.20	**0.56** ^c^
Eigenvalue		3.20	1.95
The proportion of total variance (%)		44.13	25.60

^a^Data with a normal distribution are presented as mean (standard deviation), while the skewed data were expressed as the median (quartile range)

^b^Values in boldface were major factor loadings that contribute to identify Factor 1

^c^Values in boldface were major factor loadings that contribute to identify Factor 2

### Serum FA pattern and BP levels

Adjusted mean of SBP and DBP with 95 % CI by tertile groups of the generated FA-factor scores was presented in Table 4. After adjustment for age, gender, BMI, and hypertension treatment, SBP and DBP levels were significantly increased in participants in the upper tertiles of Factor 1 scores (low LA/high SFA pattern: 14:0, 16:0, 16:1n-7, 18:2n-6, 18:3n-6) compared with those in the bottom tertiles (*P* for trend = 0.006 and 0.019, respectively). Additional adjustment for lifestyle factors (model 1) did not obviously alter theses results (*P* for trend < 0.007 and = 0.038, respectively). Even after additional adjustment for family history of hypertension, HR, TC, TG, Fbg levels (model 2), the association remained significant, with each a SD increment of Factor 1 score associated with an increment of 2.44 (95 % CI: 1.73, 3.15) mm Hg for SBP and 1.46 (95 % CI: 0.70, 2.22) mm Hg for DBP, respectively. By contrast, participants in the upper tertiles of Factor 2 scores (n-3 PUFA pattern: 20:5n-3, 22:5n-3, 22:6n-3 and 18:1n-9) had significantly decreased DBP levels compared with those in the bottom tertiles (*P* for trend = 0.026). The association slightly attenuated but remain significant in lifestyle-adjusted model 1 (*P* for trend = 0.042). The results with additional adjustment for clinical factors in model 2 were similar to those of lifestyle-adjusted model 1 (*P* for trend = 0.037), and per a SD increment of Factor 2 scores was associated with a reduction of 1.40 (95 % CI: 0.80, 2.041) mm Hg for DBP.

**Table 4:** Adjusted blood pressure mean with 95 % confidence interval by tertiles of FA-factor scores in 2447 individuals

Factor scores (Dominant FA)	*n*		Adjusted SBP Mean (95 % CI)			Adjusted DBP Mean (95 % CI)	
		Initial model^a^	Model 1^b^	Model 2^c^	Initial model^a^	Model 1^b^	Model 2^c^
Factor 1(14:0, 16:0, 16:1n-7, 18:2n-6, 18:3n-6, 20:3n-6)							
T_1_ (<−0.46)	832	123.51 (123.07, 124.00)	123.48 (122.97, 124.01)	123.80 (122.26, 124.33)	80.30 (80.12, 80.57)	80.22 (79.85, 80.49)	80.33 (80.06, 80.61)
T_2_ (−0.46–0.30)	783	126.81 (126.31, 127.39)	126.89 (126.34, 127.45)	127.30 (126.30, 127.91)	81.23 (80.02, 81.44)	81.34 (81.07, 81.69)	81.60 (81.30, 81.89)
T_3_ (≥ 0.30)	832	130.33 (129.81, 130.88)	130.55 (129.98, 132.23)	131.11 (130.48, 131.75)	82.12 (81.81, 82.33)	82.24 (81.98, 82.54)	82.58 (82.27, 82.98)
*P* for trend^d^		0.006	0.007	0.016	0.019	0.038	0.030
Factor 2 (20:5n-3, 22:5n-3, 22:6n-3, 18:1n-9)							
T_1_ (<−0.35)	831	129.72 (129.17, 130.26)	128.05 (127.46, 128.62)	127.80 (127.28, 128.32)	82.25 (82.05, 82.44)	82.27 (82.02, 82.53)	82.99 (82.59, 83.20)
T_2_ (−0.45–0.38)	807	126.87 (126.28, 127.45)	126.57 (126.01, 127.12)	126.43 (125.94, 126.92)	81.05 (80.86, 81.24)	81.03 (80.78, 81.29)	81.24 (80.95, 81.53)
T_3_ (≥ 0.38)	809	125.87 (125.34, 126.40)	126.36 (125.81, 126.90)	126.40 (125.91, 126.88)	80.12 (80.05, 80.34)	80.46 (80.20, 80.72)	79.84 (79.29, 80.35)
*P* for trend^d^		0.101	0.782	0.804	0.026	0.042	0.037

*FA* fatty acid, *n* number of subjects, *SBP* systolic blood pressure, *DBP* diastolic blood pressure, *CI* confidence interval, *T*_*3*_ the upper tertiles, *T*_*1*_ the bottom tertiles

^a^Initial model was adjusted for age, gender, BMI and hypertension

^b^Model 1 was adjusted for covariates in the initial model plus smoking, alcohol intake, education, profession, exercise, and salt intake

^c^Model 2 was adjusted for covariates in the model 1 plus family history, heart rate, triglyceride, total cholesterol and fasting blood-glucose level

^d^ *P* for trend was estimated by a multiple regression model, with ordinal numbers 0–2 assigned to tertile categories of each fatty acid

### The major individual FA and BP levels

Adjusted mean of SBP and DBP with 95 % CI by tertile groups of individual FA composition in serum was presented in Additional file 1: Table S1. After controlling for age, gender, BMI and hypertension treatment, SBP and DBP levels significantly increased with increasing in serum proportions of 16:0, 16:1n-7, 18:1n-9 and 18:3n-6 (all *P* for trend <0.05), whereas SBP significantly decreased only with 18:2n-6 increasing (*P* for trend <0.001). Decreased DBP levels were associated with increased proportions of 20:5n-3 and 22:6n-3 (*P* for trend = 0.017 and 0.010, respectively). Additional adjustment for smoking, drinking, high-salt diet, education and profession (model 1) did not appreciably alter these associations, except for 18:1n-9. The results with additional adjustment for family history, TC, TG, HR and Fbg levels (model 3) were similar to those of model 2. A significant positive association of SBP was observed with 16:0, 16:1n-7 and 18:3n-6 (*P* for trend = 0.034, 0.006 and 0.024, respectively), whereas an inverse association was observed for 18:2n-6 (*P* for trend = 0.021). Both SBP and DBP levels were significantly decreased in participants with the top tertiles of 22:6n-3 compared with those with the bottom tertiles (*P* for trend = 0.032 and 0.024, respectively).

## Discussion

In the present study, serum FA profile in hypertensive participants has been found to be typically characterized by higher 14:0, 16:0, 16:1n-7, 18:3n-6, 20:3n-6 and Δ^6^-desaturase index (18:3n-6/18:2n-6) as well as lower 18:2n-6, 22:6n-3 and Δ^5^-desaturase index (20:4n-6/20:3n-6) compared with normotensive participants. Factor analyses showed that Factor 1 (low LA/high SFA pattern: 14:0, 16:0, 16:1n-7, 18:3n-6, 18:2n-6) and Factor 2 (n-3 PUFA pattern: 20:5n-3, 22:5n-3, 22:6n-3, 18:1n-9) were identified as an indicator of the serum FA pattern. Serum low LA/high SFA pattern was adversely associated with both SBP and DBP, whereas n-3 PUFA pattern was beneficially associated with DBP. These associations cannot be apparently altered even after adjustment for age, gender, BMI, hypertension, lifestyle and clinical factors. Such findings further build and extend on the relationship between serum FA as biomarker and BP levels in Chinese.

A prior cross-sectional study of 1081 urban Italian women aged 20–69 years [18] and a large prospective cohort study of 4652 subjects aged 35–63 years from France [19] suggested that no significant association was found between intake of n-3 PUFA and BP levels. A possible explanation would be underestimated association due to diet assessment errors and recall bias during a dietary questionnaire. In contrast, circulating levels of individual FA can reflect both dietary consumption and relevant biological processes, which can be regarded as a helpfully complementary tool for food frequency questionnaire (FFQ) to closely assess diet intakes. Previous studies of FA biomarkers suggested that increased BP levels were associated with a high content of SFA and low n-3 PUFA in blood [11, 20, 21]. Nevertheless, to date, data of serum/plasma FA in relation to BP levels of individuals are scarce in China. Accumulating evidence has showed that a serum FA pattern characterized by high proportions of 16:0, 16:1n-7, 20:3n-6 and low 18:2n-6 was associated with stroke [22] and left ventricular hypertrophy [23]. In agreement with the previous findings, we found that the serum low LA/high SFA pattern was strongly associated with increased SBP. It is known that dyslipidemia and BMI were associated with greater BP levels [24, 25]. Increased levels of blood TG can damage endothelial function mediated by nitric oxide production as well as accelerate structural changes in large arteries [26]. A higher BMI can increase BP through elevated cardiac output, expanded blood volume, and boosted peripheral vascular resistance [27]. However, the positive association of low LA/high SFA pattern with BP levels did not apparently altered even after additional adjustment for these cardiometabolic risk factors in the present study. Accordingly, we speculated that the adverse effects might be attributable to the main contribution made by individual 16:0, 16:1n-7 and 18:3n-6. The multivariable analysis of tertile groups of individual FA showed that SBP levels trend to increase as increasing in serum composition of 16:0, 16:1n-7 and 18:3n-6, which was concordant with results from many investigators of circulating individual FA in relation to BP [9, 10, 28]. Serum SFA and MUFA may be highly correlated with dietary intakes of saturated fat in persons that consumed a high-SFA diet [7]. Circulating levels of 18:3n-6 trend to increase on a high-SFA diet, possibly because dietary saturated FA can influence enzymes involved in its metabolism [29]. Thus, the serum FA pattern characterized by high 16:0, 16:1n-7, 18:3n-6 and 20:3n-6 as well as low 18:2n-6 perhaps reflected a diet high in saturated fat relative to polyunsaturated fat in this middle-aged and elderly Chinese population.

Current American Heart Association dietary guidelines for CVD prevention in the community recommends an increase in both n-6 and n-3 PUFA consumption together with a reduction in SFA. A cross-sectional study conducted in Guangzhou, China has reported that erythrocyte compositions of long-chain n-3 PUFA was associated with reduction of BP levels in a general Chinese population [12]. In line with the study, our data indicated that participants in the upper tertiles of serum 22:6n-3 had significantly decreased BP levels compared with those in the bottom tertiles, which cannot be altered even by multiple adjustments. We therefore still suggest that higher intakes of food n-3 FA will increase exposure in vivo, which can favorably influence BP levels for Chinese persons. Nevertheless, the beneficial impacts by serum n-3 PUFA pattern were not observed on SBP levels with additional adjustment for possible confounders. We also found that no significant association of BP with individual 18:1n-9 in the multivariate-adjusted model. Thus, a possible explanation was that the lower-SBP effects by n-3 PUFA may be offseted by a strongly negative determinant from 18:1n-9 in n-3 PUFA factor. These observed associations together support that the key to dietary fat consumption benefits for BP levels lies not only in supplementations of its quantity, but also in the improvement in its quality.

There is a large number of evidence to suggest that the opposing effects of dietary saturated fat and polyunsaturated fat on BP levels. Dietary saturated fat can adversely influence BP levels by increasing oxidative stress and decreasing nitric oxide (NO) production within the endothelium [30]. Alterations in the metabolism of PUFAs might be a predisposing factor to the development of essential hypertension [31–33]. A case-control study of plasma phospholipids FA profile analysis revealed that higher 16:0, 18:0, 18:3n-6 and lower 18:2n-6 were found in hypertensive patients compared to normal controls [33]. Similar to results from the study, a significant difference in serum FA profile was found between hypertensive and normotensive individuals in the present study, and we also found that Δ^6^-desaturase index was markedly higher, but Δ^5^-desaturase index was lower in hypertension patients compared with normotensive persons, suggesting that essential FA and their metabolites may play an important role in the pathobiology of hypertension [34]. Our study indicated that the hypertensive patients consumed lower 18:2n-6 than normal, which was supported by a study by McCarron et al [35]. 18:2n-6 can be converted to 18:3n-6 by Δ^6^-desaturase in human body, which may be modified by dietary nutrients (e.g., magnesium) as cofactors [32]. 20:5n-3 can block the activity of Δ^5^-desaturase such that 20:4n-6 levels in tissue will be low. 20:4n-6 can form the precursor of 2-series of PGs that stimulates vascular constriction and smooth muscle contraction, whereas 20:5n-3 is precursor of PGI_3_ and thromboxane A_3_ that have been reported to be less active. Thus, increased consumption of marine food rich in 20:5n-3 and 22:6n-3 can competitively inhibit hepatic Δ^5^-desaturase to reduce circulating levels of metabolites from 20:4n-6 such as PGF_2a_and thromboxane A_2_ [36]. Possible mechanisms whereby n-3 FA may favorably influence BP may be multifactorial involving improvements in endothelial vasodilator function [37, 38], reactivity of resistant vessel vascular smooth muscles [39] and arterial compliance [40], along with a cardiac effect mediated by a decrease in heart rate to provide an energy-sparing promotion of diastolic relaxation [41]. In addition, BP and cardiac β-adrenergic receptor responsiveness decreased on a low-fat diet with a high ratio of PUFA/SFA (P/S) [42]. However, the P/S ratio and total dietary fat are often altered in diet, and thus the oppositive mechanisms between saturated fat and polyunsaturated fat have not yet been understood and remain to be further elucidated.

Dietary nutrients as cofactors are necessary for the formation of potent platelet anti-aggregators and vasodilators such as prostaglandin E1 (PGE1) and PGI_3_, which can prevent the development of hypertension [32]. Lower magnesium can reduce the Δ 6-desaturase activity, leading to an inadequate the conversion of 20:3n-6 to PGE1 [43]. Vitamin C can improve nitric oxide (NO) production and enhance the synthesis of PGE1 in hypertension [44, 45]. Many studies found that higher intake of salt can lead to BP, but sodium seems to act in concert with other nutrients such as calcium, potassium, magnesium, vitamin C, and antioxidants present in fruits and vegetables act together to determine the ultimate degree of BP by regulating the release of PGE1, PGI_2_ and NO production [32, 46]. We found that higher intake of salt was present in hypertension patients compared with normotensive participants, which was supported by the observation that plasma NO levels decreased in patients with hypertension after salt loading [47], suggesting that excess salt intake can reduce NO production from the endothelial cells in patients with salt-sensitive hypertension. However, we was unable to exclude the effects of total energy intake or the consumption of PUFAs, saturated fats, trans-fats and other food nutrients on BP in the study population, as dietary intakes were not assessed at baseline. A dietary survey of middle-aged nutrient intake in East (China and Japan) Asian and Western (UK and US) persons revealed that Chinese sample population has lower intake of total fat, saturated fat, trans-fatty acid, potassium, vitamin C and calcium as well as higher sodium and sodium/potassium ratio compared to western persons, whereas no marked difference in dietary intake of magnesium were found between the two populations [48]. In addition, peroxidation of unsaturated FA such as 18:3n-3 can result in the formation of aldehydes such as 4-hydroxypentenal causing both hypertension and oxidative stress. Superoxide anion can inactivate PGI_2_ and NO [49], which can lead to elevated BP by an increase in peripheral vascular resistance. Evidence indicated that the superoxide dismutase activity and vitamin E concentration were found to be lower, whereas plasma superoxide anion and lipid peroxides were found to be higher in patients with uncontrolled hypertension [44, 50]. Thus, the interaction among free-radical, NO, PUFAs, antioxidants and other dietary nutrients on BP in the study population may be helpful for understanding the aetiopathogenesis of hypertension, but its mechanism is presently unclear [51, 52]. Further studies were merited to clarify the biological plausibility related to PUFAs interaction with free radicals, NO and other nutrients in the development of hypertension.

Many a strengths should be highlighted in our study. Firstly, we used a method of exploratory factor analyses to identify the major FA-factors as indicators of serum FA patterns, facilitating the interpretation of the findings. Additionally, the amount of exogenous and endogenous FA can be closely represented by serum FA, independent of diet records and recalls bias. However, the potential limitations were likely to bias the endings. Firstly, the causal relations cannot be determined. Secondly, we cannot affirm that a dietary pattern of low LA/high SFA can unfavorably influence BP levels, because data are lacking on food FA and BP. Thirdly, the part of the positive association between BMI and BP may depend on dietary fat intakes, considering that BMI is a strong determinant of BP levels. It may be questioned whether controlling for BMI in the multivariate model is appropriate when assessing the strength of the linear association. Fourthly, all participants were recruited from the communities in Zhejiang province located in southeast China, which did not perfectly represent a random sample of a general Chinese population. Thus, caution is required in generalizing the current results to the whole middle-aged and elderly Chinese. Fifthly, participants with prevalent hypertension were identified by self-reported information on anti-hypertensive drugs use, which might be subject to report bias. Sixthly, the clear association between FA-factors and BP can be drawn in the present study, possibly because limited community-dwellers living in the same regions are those who have a similar life style. Thus, it is necessary to further demonstrate whether these effects of individual FA on BP can be general in Chinese adults. Finally, we cannot exclude the possibility that the unknown or incompletely measured valuables modified the findings.

## Conclusions

In conclusion, the serum FA pattern characterized by low proportions of 16:0, 16:1n-7 and 18:3n-6 as well as high 18:2n-6 and 22:6n-3 was associated with reduced BP levels in this middle-aged and elderly Chinese population. Such evidence well supports the current dietary recommendations in the communities to replace saturated fat with polyunsaturated fat. A well-designed cohort study is further needed to prospectively confirm the effects of individual FA in circulating blood on BP levels in a larger Chinese population.

## Additional file

Additional file 1: Table S1. Adjusted blood pressure mean with 95 % confidence interval by tertiles of the major serum FA in subjects (*n* = 2447). (DOC 132 kb)

## Abbreviations

FA: fatty acid
BP: blood pressure
PUFA: polyunsaturated fatty acid
MUFA: monounsaturated fatty acid
LC: long-chain
BMI: body mass index
HR: heart rate
SBP: systolic blood pressure
DBP: diastolic blood pressure
TG: Triglycerides
TC: total cholesterol
Fbg: fasting blood-glucose levels
GLC: gas-liquid chromatography
CI: confidence interval
FFQ: food frequency questionnaire
SFA: Saturated fatty acid
LA: linoleic acid
NO: nitric oxide
P/S: PUFA/SFA
