# Supplementary material for: Exploratory serum fatty acid patterns associated with blood pressure in community-dwelling middle-aged and elderly Chinese
Source: Lipids Health Dis. 2016 Mar 18;15:58. doi: 10.1186/s12944-016-0226-3 (PMC4797152; doi:10.1186/s12944-016-0226-3)
Supplement: Additional file 1: Table S1. — Adjusted blood pressure mean with 95 % confidence interval by tertiles of the major serum FA in subjects (n = 2447). (DOC 132 kb) [file 12944_2016_226_MOESM1_ESM.doc]

| **Table S1. Adjusted blood pressure mean with 95% confidence interval** **by tertiles of the major serum FA in 2447 individuals** | | | | | | | | |
| --- | --- | --- | --- | --- | --- | --- | --- | --- |
| Major FA | n |  | Adjusted SBP Mean (95% CI) |  |  |  | Adjusted DBP Mean (95% CI) |  |
|  |  | Initial model^a^ | Model 1^b^ | Model 2^c^ |  | Initial model^a^ | Model 1^b^ | Model 2^c^ |
| 14:0 |  |  |  |  |  |  |  |  |
| T_1_ (2.60-19.59) | 816 | 125.73 (125. 17, 126.29) | 125.60 (125.10, 126.11) | 125.75 (125. 17, 126.32) |  | 80.91 (80.67, 81.14) | 80.92 (80.67, 81.16) | 80.91 (80.62, 81.19) |
| T_2_ (19.59-21.68) | 812 | 127.30 (126.32, 127.78) | 127.06 (126.53, 127.58) | 127.36 (126.78, 127.94) |  | 81.38 (81.13, 81.62) | 81.44 (81.18, 81.69) | 81.85 (80.54, 82.15) |
| T_3_ (21.68-29.70) | 819 | 128.16 (127.59, 128.74) | 127.89 (127.36, 128.42) | 128.06 (127.50, 128.64) |  | 81.48 (81.24, 81.72) | 81.61 (81.37, 81.87) | 82.03 (81.75, 82.32) |
| *P* for trend^d^ |  | 0.053 | 0.136 | 0.094 |  | 0.535 | 0.481 | 0.199 |
| 16:0 |  |  |  |  |  |  |  |  |
| T_1_ (2.60-19.59) | 811 | 124.75 (123.57, 125.94) | 124.05 (123.48, 124.63) | 124.00 (123.40, 124.62) |  | 80.33 (80.16, 80.52) | 79.47 (79.17, 79.77) | 79.45 (79.15, 79.75) |
| T_2_ (19.59-21.68) | 812 | 126.29 (125.08, 127.51) | 126.47 (125.99, 127.05) | 126.53 (126.01, 127.06) |  | 81.30 (81.12, 82.48) | 80.40 (80.24, 80.75) | 80.51 (80.24, 80.79) |
| T_3_ (21.68-29.70) | 824 | 129.56 (128.33, 130.79) | 128.98 (129.46, 128.50) | 128.96 (128.44, 129.48) |  | 82.15 (81.97, 82.33) | 81.64 ( 81.39, 81.98) | 81.64 (81.38, 81.91) |
| *P* for trend^d^ |  | <0.001 | 0.022 | 0.034 |  | 0.020 | 0.058 | 0.076 |
| 16:1n-7 |  |  |  |  |  |  |  |  |
| T_1_ (0.20-1.10) | 804 | 123.87 (123.64, 124.10) | 123.31 (122.78, 123.84) | 123.29 (122.72, 123.58) |  | 80.41 (80.23, 80.59) | 79.48 (79.20, 79.75) | 79.46 (79.17, 79.74) |
| T_2_ (1.10-1.78) | 827 | 126.97 (126.75, 127.19) | 127.00 (126.52, 127.46) | 127.04 (127.04, 127.57) |  | 81.37 (81.19, 81.55) | 80.73 (80.46, 81.01) | 80.78 (80.48, 81.06) |
| T_3_ (1.78-5.74) | 816 | 129.74 (129.41, 129.96) | 129.63 (129.15, 130.10) | 129.60 (129.06, 130.14) |  | 82.02 (81.84, 82.20) | 81.57 (81.30, 81.84) | 81.55 (81.27, 81.83) |
| *P* for trend^d^ |  | <0.001 | 0.008 | 0.006 |  | 0.019 | 0.169 | 0.194 |
| 18:1n-9 |  |  |  |  |  |  |  |  |
| T_1_ (6.49-17.43) | 814 | 124.85 (124.63, 125.08) | 125.33 (124.94, 125.71) | 125.38 (124.98, 125.78) |  | 79.97 (79.34, 80.59) | 80.51 (80.31, 80.71) | 80.53 (80.322, 80.73) |
| T_2_ (17.43-20.77) | 813 | 126.83 (126.60, 127.05) | 127.16 (126.81, 127.52) | 127.08 (126.71, 127.45) |  | 81.90 (81.23, 82.57) | 81.27 (81.08, 81.46) | 81.23 (81.03, 81.44) |
| T_3_ (20.77-34.71) | 820 | 128.92 (128.70, 129.15) | 128.69 (128.36, 129.02) | 128.72 (128.36, 129.09) |  | 81.78 (81.12, 82.45) | 82.16 (81.97, 82.35) | 82.18 (81.99, 82.38) |
| *P* for trend^d^ |  | <0.001 | 0.09 | 0.252 |  | 0.008 | 0.069 | 0.225 |
| 18:2n-6 |  |  |  |  |  |  |  |  |
| 10.44-25.84 | 814 | 129.87 (129.64, 130.10) | 129.70 (129.38, 130.01) | 129.77 (129.17, 130.38) |  | 81.87 (81.69, 82.05) | 81.06 (80.77, 81.35) | 81.08 (80.77, 81.39) |
| 25.84-30.48 | 815 | 127.06 (126.84, 127.28) | 127.43 (127.10, 127.76) | 127.32 (126.79, 127.85) |  | 81.48 (81.30, 81.65) | 80.91 (80.64, 81.19) | 80.88 (80.59, 81.16) |
| 30.48-45.03 | 818 | 123.73 (123.52, 123.94) | 124.00 (123.64, 124.35) | 123.91 (123.40, 124.41) |  | 80.44 (80.26, 80.62) | 80.04 (79.78, 80.29) | 80.06 (79.80, 80.32) |
| *P* for trend^d^ |  | <0.001 | 0.010 | 0.021 |  | 0.051 | 0.635 | 0.714 |
| 18:3n-6 |  |  |  |  |  |  |  |  |
| 0.04-0.25 | 815 | 125.03 (124.81, 125.28) | 125.42 (125.03, 125.80) | 125.45 (125.02, 125.84) |  | 80.60 (80.42, 80.78) | 80.83 (80.62, 81.03) | 80.84 (80.63, 81.06) |
| 0.25-0.42 | 817 | 127.12 (126.89, 127.35) | 127.47 (127.11, 127.83) | 127.45 (127.06, 127.88) |  | 81.47 (81.30, 81.64) | 81.56 (81.37, 81.76) | 81.53 (81.32, 81.74) |
| 0.42-1.29 | 815 | 128.48 (128.24, 128.72) | 128.38 (128.04, 128.71) | 128.40 (128.01, 128.78) |  | 81.72 (81.54, 81.89) | 81.60 (81.41, 81.78) | 81.62 (81.42, 81.82) |
| *P* for trend^d^ |  | <0.001 | 0.021 | 0.024 |  | 0.011 | 0.157 | 0.150 |
| 20:4n-6 |  |  |  |  |  |  |  |  |
| 1.33-5.24 | 810 | 127.44 (126.90, 127.98) | 127.53 (126.98, 128.08) | 127.81(127.22, 128.40) |  | 80.82 (80.61, 81.03) | 80.81 (80.58, 81.04) | 81.00 (80.75, 81.26) |
| 5.24-6.65 | 816 | 126.41 (125.88, 126.94) | 126.53 (125.99, 127.06) | 127.04 (126.45, 127.62) |  | 81.09 (80.90, 81.29) | 81.16 (80.93, 81.39) | 81.20 (80.97, 81.50) |
| 6.65-11.90 | 821 | 125.20 (124.66 125.72) | 125.02 (124.48 125.57) | 125.15 (124.58, 125.72) |  | 81.36 (81.16, 81.59) | 81.35 (81.11, 81.60) | 81.61(81.34, 81.87) |
| *P* for trend^d^ |  | 0.082 | 0.064 | 0.106 |  | 0.119 | 0.261 | 0.320 |
| 20:5n-3 |  |  |  |  |  |  |  |  |
| 0.12-2.32 | 802 | 126.31 (125.76, 126.85) | 126.29 (125.73, 126.84) | 126.52 (125.92, 127.12) |  | 81.83 (81.72, 81.93) | 81.95 (81.76, 82.15) | 81.94 (81.73, 82.15) |
| 2.32- 3.71 | 804 | 126.29 (125.76, 126.82) | 126.34 (125.80, 126.88) | 126.73 (126.12, 127.32) |  | 81.26 (81.16, 81.47) | 81.28 (81.07, 81.50) | 81.31 (81.09, 81.54) |
| 3.71-8.88 | 841 | 126.69 (126.15, 127.24) | 126.71 (126.14, 127.28) | 126.96 (126.36, 127.57) |  | 80.65 (80.34, 80.81) | 80.80 (80.60, 81.02) | 80.78 (80.57, 80.99) |
| *P* for trend^d^ |  | 0.702 | 0.849 | 0.483 |  | 0.017 | 0.048 | 0.061 |
| 22:5n-3 |  |  |  |  |  |  |  |  |
| 0.05-0.42 | 817 | 126.69 (126.15, 127.24) | 127.30 (127.18, 127.42) | 127.66 (127.06, 128.25) |  | 80.92 (80.71, 81.12) | 80.86 (80.60, 81.22) | 81.19 (80.93, 81.46) |
| 0.42-0.53 | 815 | 126.36 (125.83, 126.90) | 126.41 (126.27, 126.55) | 126.54 (125.95, 127.14) |  | 81.06 (80.86, 81.27) | 81.18 (80.94, 81.42) | 81.31 (81.05, 81.56) |
| 0.53-2.50 | 815 | 126.23 (125.70, 126.77) | 125.02 (124.84, 125.20) | 125.49 (124.91, 126.07) |  | 81.27 (81.07, 81.58) | 81.27 (81.30, 80.51) | 81.35 (81.09, 81.60) |
| *P* for trend^d^ |  | 0.361 | 0.138 | 0.072 |  | 0.672 | 0.714 | 0.875 |
| 22:6n-3 |  |  |  |  |  |  |  |  |
| 0.22-1.38 | 815 | 127.80 (127.24, 128.35) | 127.89 (127.30, 128.48) | 128.56 (128.25, 128.86) |  | 81.97 (81.56, 82.08) | 82.00 (81.69, 82.28) | 82.30 (82.04, 82.57) |
| 1.38-1.92 | 816 | 126.64 (126.09, 127.19) | 126.32 (125.74, 126.91) | 126.40 (126.09, 126.71) |  | 81.17 (80.96, 81.48) | 81.14 (80.90, 81.39) | 81.30 (81.04, 81.56) |
| 1.92-4.32 | 816 | 124.91 (124.40, 125.24) | 125.11 (124.57, 125.65) | 125.39 (125.14, 125.65) |  | 80.25 (80.04, 80.45) | 80.29 (81.06, 80.50) | 80.37 (80.13, 80.61) |
| *P* for trend^d^ |  | 0.029 | 0.044 | 0.032 |  | 0.010 | 0.017 | 0.024 |
| FA, fatty acid; n, number of subjects; SBP, systolic blood pressure; DBP, diastolic blood pressure; CI, confidence interval; T_3_, the upper tertiles; T_1_, the bottom tertiles.  ^a^ Initial model was adjusted for age, gender, BMI and hypertension.  ^b^ Model 1 was adjusted for covariates in the initial model plus smoking, alcohol intake, education, profession, exercise, and salt intake.  ^c^ Model 2 was adjusted for covariates in the model 1 plus family history, heart rate, triglyceride, total cholesterol and fasting blood-glucose level.  ^d^ *P* for trend was estimated by a multiple regression model, with ordinal numbers 0-2 assigned to tertile categories of each fatty acid. | | | | | | | | |
